# Co-Fermentation of Trichoderma Strains as a Biotechnological Strategy to Enhance Enzyme Production and Plant Growth-Promoting Metabolites for Agricultural Applications

**DOI:** 10.3390/microorganisms14071524

**Published:** 2026-07-13

**Authors:** Isabela L. Valente, Ádrian G. Dorneles, Giovani L. Zabot, Marcio A. Mazutti

**Affiliations:** 1 Department of Chemical Engineering, Federal University of Santa Maria (UFSM), 1000 Roraima Av., Camobi, Santa Maria 97105-340, Brazil; isabelavalentee@gmail.com (I.L.V.); adrian.guterres@acad.ufsm.br (Á.G.D.); 2 Laboratory of Agroindustrial Process Engineering (LAPE), Federal University of Santa Maria (UFSM), 3012 Taufik Germano Rd., Cachoeira do Sul 96503-205, Brazil; giovani.zabot@ufsm.br

**Keywords:** agricultural biotechnology, bio-based inputs, enzyme production, microbial biocatalysis, plant growth-promoting metabolites, *Trichoderma* co-fermentation

## Abstract

The growing demand for sustainable agricultural inputs has encouraged the development of biotechnological alternatives based on microbe-derived enzymes and bioactive metabolites. In this study, co-fermentation strategies involving different *Trichoderma* strains were investigated as a process-based approach to enhance the production of enzymes and plant growth-promoting metabolites with agricultural relevance. Distinct culture media compositions and inoculation strategies significantly affected microbial performance and biocatalytic outputs. Optimized co-culture conditions resulted in enhanced conidiation, microsclerotia formation, siderophore production (up to 95%), phosphate solubilization (up to 419 mg mL^−1^), indole-3-acetic acid synthesis (0.60 mg mL^−1^), and increased activities of chitinase, β-1,3-glucanase, and protease. Metabolomic profiling by GC-MS revealed the induction of diverse secondary metabolites associated with antimicrobial activity and plant–microbe signaling. Overall, the results demonstrate that *Trichoderma* co-fermentation is an effective biotechnological strategy to intensify enzyme and metabolite production, highlighting its potential for the development of multifunctional bioinputs for sustainable agricultural applications.

## 1. Introduction

The increasing demand for sustainable agricultural practices has intensified interest in biological strategies that enhance nutrient cycling, suppress soil-borne pathogens, and improve plant performance. Among beneficial microorganisms, fungi of the genus *Trichoderma* have gained prominence due to their ability to antagonize phytopathogens, stimulate plant growth, and improve nutrient availability. These fungi are cosmopolitan, fast-growing, and metabolically versatile, producing a wide spectrum of hydrolytic enzymes, siderophores, phytohormones, and secondary metabolites. They represent a promising alternative to agrochemicals (pesticides, larvicides, fungicides, herbicides, and others), which cause multiple adverse effects on soil health, human health, and the environment, and contribute to the development of pest resistance [1,2].

Endophytic fungi are metabolically active microorganisms that establish mutualistic interactions with their host plants and are capable of activating metabolic pathways while persisting within plant tissues. These microorganisms are considered a favorable natural resource for field applications due to their extensive biodiversity and their ability to produce large amounts of biologically active compounds, which enable plants to adapt to their natural habitats [3]. Endophytic fungi colonizing plants predominantly belong to the phylum Ascomycota or to mitosporic fungi, with some representatives from the phyla Oomycota, Basidiomycota, and Zygomycete fungi [4].

*Trichoderma* is a genus of Hypocrea fungi belonging to the phylum Ascomycota, cosmopolitan in distribution and widely used in agriculture and in the biological control of phytopathogens [5]. Species of this genus are found in a wide range of environments, including soils and rhizospheres, decaying wood, sediments, and various organic substrates. *Trichoderma* is considered a promising fungus in both industrial and agricultural sectors due to its ability to produce enzymes and antibiotics, promote plant growth, induce systemic resistance, and enhance nutrient uptake [6].

In addition to their ecological relevance, *Trichoderma* species have gained increasing attention in biocatalysis and agricultural bioprocessing due to their capacity to produce a wide range of hydrolytic enzymes and bioactive metabolites under controlled fermentation conditions [7]. From a bioprocess perspective, the modulation of nutritional and operational parameters represents a key strategy to intensify enzyme yields and metabolite synthesis, enabling the development of scalable bio-based products for agricultural applications [8,9].

Co-culture systems involving two compatible strains may provide a more effective mode of action than single-strain applications, thereby promoting higher stability in biological control strategies [10]. Studies have shown that co-fermentation of *Pseudomonas fluorescens* with *Trichoderma asperellum* results in a higher reduction in infections caused by *Rhizoctonia solani* and *Fusarium oxysporum*, as well as increased cotton plant growth [10]. Similarly, co-cultivation of *T. atroviride* with *Bacillus amyloliquefaciens* exhibited enhanced biocontrol efficacy in wheat crops against *Fusarium graminearum*, in addition to increased enzyme synthesis and activation of signaling molecules [11].

Furthermore, co-inoculation of the nitrogen-fixing bacterium *Bradyrhizobium* spp. with the arbuscular mycorrhizal fungus *Glomus mosseae* promotes higher plant biomass production in nitrogen- and phosphorus-deficient soils cultivated with soybean. In line with these findings, co-culture of *B. japonicum* and *T. harzianum* resulted in functional nodulation in soybean crops, along with improved biocontrol activity [12]. Co-fermentation of the fungus *Pestalotia* spp. with a marine bacterium led to the discovery of a novel antibiotic, pestalone. Moreover, co-cultivation of the endophytic fungus *Chaetomium* spp. with the bacterium *Bacillus subtilis* promoted higher production of secondary metabolites compared with monocultures in rice fields [13].

Therefore, the objective of this study was to evaluate co-fermentation strategies of different *Trichoderma* strains as a biotechnological approach to enhance the production of enzymes and plant growth-promoting metabolites. The influence of inoculation timing, strain proportion, and culture medium composition on key biocatalytic outputs, such as phosphate solubilization, siderophore and indole-3-acetic acid production, hydrolytic enzyme activities, and secondary metabolite profiles, was systematically assessed, aiming at the development of multifunctional bio-based inputs for agricultural applications.

## 2. Materials and Methods

### 2.1. Microbial Strains

The *Trichoderma harzianum* strain IB 19/17 was provided by the Biological Institute of Campinas (Campinas, SP, Brazil). The *Trichoderma harzianum* strain MMBF 58/09 was provided by the Biological Institute of São Paulo, and the *Trichoderma asperellum* strain URM 6997/160821 was provided by the Bioprocesses Laboratory of the Federal University of Santa Maria (UFSM, Santa Maria, Brazil). The *Trichoderma* strains were cultivated on Potato Dextrose Agar (PDA) (EP/USP/BAM, KASVI) at 25 °C under a photoperiod for 7 days in a photoperiod-controlled incubator (SL 224, SOLAB, Piracicaba, Brazil).

### 2.2. Compatibility Test Among Trichoderma Strains

In the compatibility test, Czapek agar medium was used (3 g L^−1^ Sodium nitrate NaNO_3_, 1 g L^−1^ dibasic potassium phosphate K_2_HPO_4_, 0.5 g L^−1^ potassium chloride KCl, 0.5 g L^−1^ magnesium sulfate heptahydrate MgSO_4_•7H_2_O, 0.01 g.L^−1^ Iron (II) sulfate heptahydrate FeSO_4_•7H_2_O, 15 gL^−1^ agar), adjusted to pH 4.8, and supplemented with 1 mL of trace metal solution (1 g Zinc sulfate heptahydrate ZnSO_4_•7H_2_O, 0.5 g Copper sulfate pentahydrate CuSO_4_•5H_2_O in 100 mL of water) with sucrose as the carbon source at 30 g·L^−1^. Spore suspensions were quantified to 5 × 10^5^ spores·mL^−1^, and inoculation was performed in three different ways: (A) 2 µL of each spore suspension from each strain were added to the plate, placed 3 cm apart (IB 19/17 + URM 6997/160821 and MMBF 58/09 + URM 6997/160821); (B) spores were mixed and pipetted into the center of the plate; and (C) 2 µL of the mixed spore suspensions were spread over the entire plate. The plates were incubated at 25 °C for 7 days or until microbial colonies interacted with each other [14].

### 2.3. Fermentation

Fermentation conditions were designed to simulate nutritional scenarios relevant to soil and rhizosphere environments, allowing the evaluation of microbial functional traits associated with soil processes. Fermentation was carried out using four different culture media, as follows: (1) 15 g·L^−1^ glucose, 10 g·L^−1^ sucrose, 2 g·L^−1^ protein hydrolysate, 3 g·L^−1^ ammonium sulfate, and pH 4.5; (2) 15 g·L^−1^ glucose, 10 g·L^−1^ sucrose, 8 g·L^−1^ protein hydrolysate, and pH 6.5; (3) 5 g·L^−1^ glucose, 2 g·L^−1^ protein hydrolysate, 3 g·L^−1^ ammonium sulfate, and pH 6.5; and (4) 10 g·L^−1^ glucose, 5 g·L^−1^ sucrose, 5 g·L^−1^ protein hydrolysate, 1 g·L^−1^ ammonium sulfate and pH 5.5. The culture media described were taken from the Plackett–Burman statistical design of the article by [15].

All experiments were conducted using a basal medium (1 g·L^−1^ potassium chloride KCl, 1.05 g·L^−1^ dibasic potassium phosphate K_2_HPO_4_, 0.36 g·L^−1^ monobasic potassium phosphate KH_2_PO_4_, 0.6 g·L^−1^ magnesium sulfate heptahydrate MgSO_4_•7H_2_O, and 0.05 g·L^−1^ glycine C_2_H_5_NO_2_), at a constant temperature of 28 °C, agitation speed of 150 rpm, and a fermentation period of 96 h in an incubator (TE-420, Tecnal, Piracicaba, Brazil) in triplicate. The spore suspension concentration was 1 × 10^6^ spores·mL^−1^ for each strain. Inoculation was performed using two strains (Co-2) at proportions of 25%, 50%, and 75%; among three strains (Co-3) at proportions of 16.7%, 33.3%, and 66.7%; and in fermentations involving two strains initially, with the addition of a third strain after 48 h (Co-48), all in equal amounts. In all cases, 1 μL of spore suspension was added per 50 mL of culture medium.

At the end of the fermentation, the analyses began with the quantification of dry biomass. The biomass was separated from the liquid medium through filtration using 125 mm filter paper and a vacuum pump(Tecnal, Piracicaba, Brazil). After being filtered, the biomass was placed in an oven at 60 °C for 24 h, then taken to a desiccator and weighed. Biomass quantification was obtained by measuring the difference in weight of the filter paper before and after filtration, once the time in the desiccator was complete.

### 2.4. Evaluation of Phosphate Solubilization, Siderophore, and IAA Production

The characterization quantified phosphate solubilization, siderophore production, and IAA production. For phosphate solubilization, the crude extract was centrifuged (5804 R, Eppendorf, Hamburg, Germany) at 5000 rpm for 10 min [16]. Phosphate solubilization was determined by adding 0.5 mL of the sample, 1 mL of the modified Murphy and Riley colorimetric reagent, and 3.5 mL of distilled water, and the mixture was allowed to stand for 20 min. Absorbance was measured at 880 nm using a spectrophotometer (UV-2600, Shimadzu, Kyoto, Japan), and quantification was performed by determining orthophosphate ions using a standard curve prepared with 80 ppm of monobasic potassium phosphate (KH_2_PO_4_) [17].

For siderophore production determination, the centrifuged crude extract was filtered using a vacuum pump, and the analysis was performed by subsequently adding the CAS solution Chrome Azurol Sulfonate to the sample at a 1:1 ratio. The mixtures were incubated for 60 min at 25 °C in the dark. Absorbance was measured at 630 nm using a spectrophotometer (UV-2600, Shimadzu, Japan), and siderophore production was calculated based on the difference between the control and the sample [18].

IAA production was determined using samples filtered using a vacuum pump. The assays were performed using Salkowski reagent (1 mL of 0.5 M FeCl_3_•6H_2_O and 50 mL of H_2_SO_4_) at a 1:1 ratio with the sample. The reaction was carried out for 30 min at 25 °C, followed by absorbance measurement at 530 nm using a spectrophotometer (UV-2600, Shimadzu, Japan), with quantification based on a standard curve prepared with IAA [17].

### 2.5. Enzymatic Activity

The assays were performed to determine chitinase, β-1,3-glucanase, and protease activities. Chitinase activity was assayed using 250 µL of 1% (*w*/*v*) colloidal chitin solution as substrate, diluted in acetate buffer, 200 µL of acetate buffer (50 mM, pH 5.0), and 50 µL of sample, incubated at 37 °C for 30 min. β-1,3-glucanase activity was determined using 1% (*w*/*v*) laminarin as substrate, diluted in acetate buffer. The assay was performed with 50 µL of enzyme solution, 250 µL of substrate solution, and sodium acetate buffer (50 mM, pH 5.0), incubated at 45 °C for 30 min.

For both chitinase and β-1,3-glucanase assays, a substrate blank and a sample blank with sodium acetate buffer (50 mM, pH 5.0) were prepared using the same proportions as in the sample analyses. After 30 min, reactions (37 °C and 45 °C, respectively) were stopped by placing the tubes in an ice bath, followed by the addition of 500 µL of 3,5-dinitrosalicylic acid. The mixtures were heated at 95 °C for 5 min and subsequently cooled again in an ice bath for 5 min. Enzymatic activity was determined at a wavelength of 540 nm using a spectrophotometer (UV-2600, Shimadzu, Kyoto, Japan). One unit of enzyme activity was defined as the amount of enzyme required to release 1 µmol of reducing sugar per minute [19,20]. N-acetyl-D-glucosamine and glucose were used to construct the standard curves for chitinase and β-1,3-glucanase, respectively.

For protease activity, the assay was performed by adding 0.5 mL of sample, 1.5 mL of substrate at a concentration of 2% (*w*/*v*) casein, diluted in phosphate buffer, and 1 mL of phosphate buffer (50 mM, pH 7.8). The reaction mixture was incubated at 30 °C for 30 min, followed by the addition of 3 mL of trichloroacetic acid (0.4 M) in an ice bath. Samples were centrifuged (5804 R, Eppendorf, Hamburg, Germany) at 5000 rpm for 10 min, and the supernatant was analyzed using a spectrophotometer (UV-2600, Shimadzu, Japan) at 280 nm [21,22]. One unit of enzymatic activity was defined as the amount of enzyme required to release 1 µg of tyrosine per milliliter per unit of absorbance per minute. Tyrosine was used in the construction of the standard curve.

### 2.6. Identification of Secondary Metabolites

The identification of secondary metabolites from the co-cultures was performed by mixing the filtered crude extract with a solvents, separately, composed of hexane, butanol, and ethyl acetate at a 1:1 ratio. The mixture was agitated for 60 s and frozen at −20 °C for 60 min. Subsequently, samples were centrifuged at 5000 rpm for 10 min, and the supernatant was subjected to GC–MS analysis [13]. A 2 µL aliquot of the extract was injected into the chromatograph–mass spectrometer system. Analyses were performed using a GC-2010 Plus gas chromatograph (Shimadzu, Kyoto, Japan) coupled to an GCMS-QP2010 Ultra mass spectrometer (Shimadzu, Kyoto, Japan), equipped with an automatic AOC-20i series injector (Shimadzu, Kyoto, Japan). Chromatographic separation was achieved using an Rtx^®^-MS capillary column (30 m × 0.25 mm, 0.25 µm film thickness) composed of 5% diphenyl and 95% dimethyl polysiloxane (Restek Corporation, Bellefonte, PA, USA). Helium was used as the carrier gas at a constant flow rate of 1.0 mL·min^−1^. The injector temperature was maintained at 280 °C, and samples were injected in splitless mode.

The oven temperature program was set to an initial temperature of 70 °C for 1 min, increased to 300 °C at a rate of 10 °C·min^−1^, and held at 300 °C for 5 min, resulting in a total chromatographic run time of 29 min. The interface and ion source temperatures were maintained at 230 °C and 300 °C, respectively. Mass spectra were acquired over an *m*/*z* range of 35–500 amu with a scan rate of 0.30 scans·s^−1^. Identification of volatile compounds was carried out using a single quadrupole mass spectrometer operating in electron impact (EI) mode at 70 eV under full-scan conditions. Individual components were identified by comparing their mass fragmentation patterns with those in the Wiley Registry of Mass Spectral Data (Palisade Corporation, Newfield, NY, USA). Only compounds with a mass spectral matching score higher than 80% were considered for further analysis.

## 3. Results

### 3.1. Compatibility Tests

**Figure 1:**
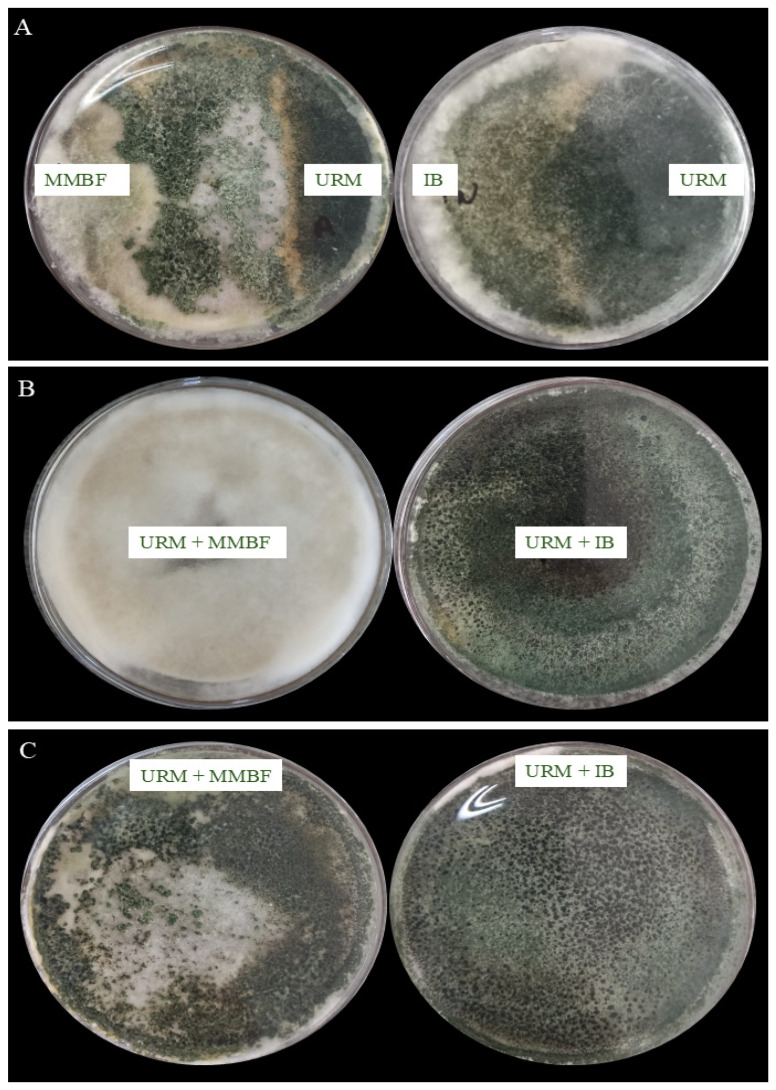
Compatibility test for the strains *T. harzianum* IB 19/17, *T. asperellum* URM 6997/160821, and *T. harzianum* MMBF 58/09: (**A**) strains placed 3 cm apart from each other (MMBF and URM on the left plate, and URM and IB on the right plate); (**B**) mixtures of the strains with inoculation at the center of the plate; (**C**) mixtures of the strains with spreading over the entire plate (MMBF + URM on the left plate, and URM + IB on the right plate).

The compatibility test was defined based on the ability of the fungi to grow without inhibition, as shown in Figure 1. For test A, the expected outcome was growth toward each other without any negative effects, indicating a positive association. In tests B and C, the expected result was that the strains would grow while sharing the same space without exhibiting inhibitory interactions. The tests showed compatible growth, with no inhibition upon contact and no excessive overgrowth of one strain over the other. Except for test B involving the mixture of the URM and MMBF strains, in which no sporulation was observed, all other tests showed compatibility.

### 3.2. Biomass and Propagule Counting

**Figure 2:**
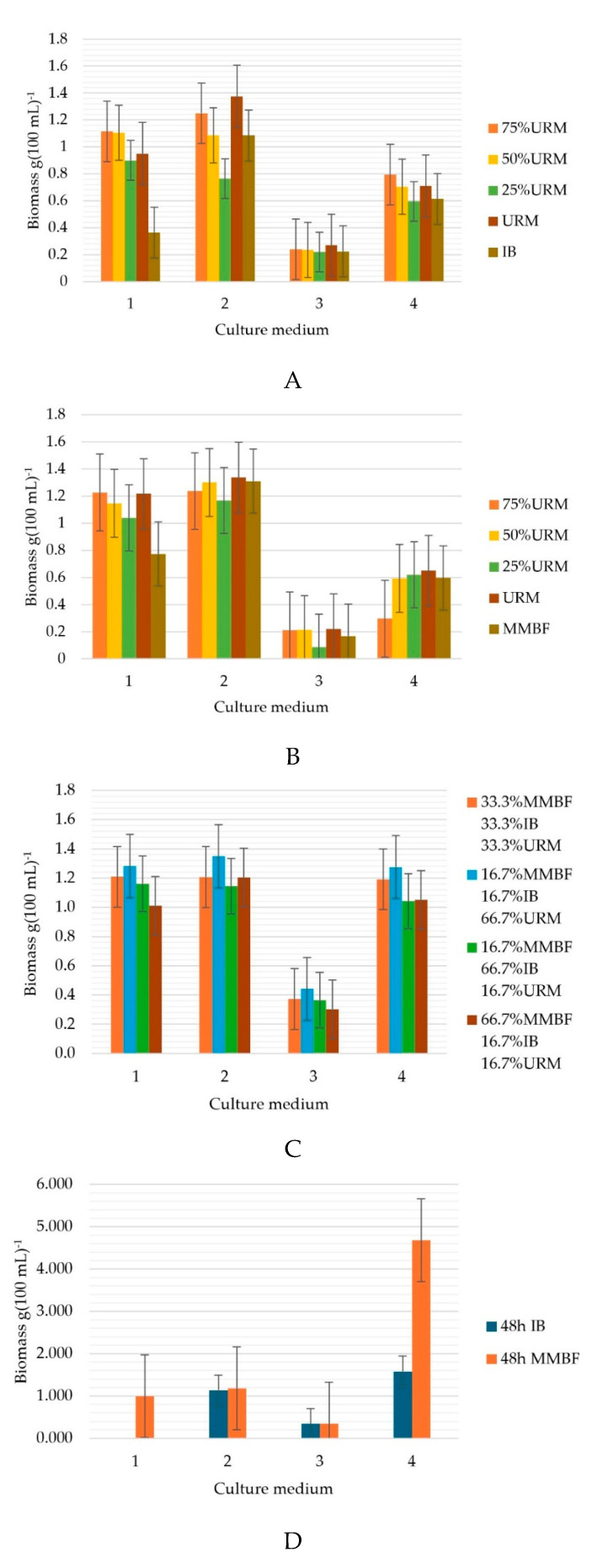
Dry biomass production: (**A**) in Co-2 with *T. harzianum* IB 19/17 and *T. asperellum* URM 6997/160821; (**B**) in Co-2 with *T. harzianum* MMBF 58/09 and *T. asperellum* URM 6997/160821; (**C**) in Co-3; and (**D**) in Co-48.

The dry biomasses obtained from the co-cultures showed the highest concentration values of 1.38 g·100 mL^−1^ for Co-2 (*T. harzianum*) (Figure 2A), 1.33 g·100 mL^−1^ for Co-2 (*T. asperellum*) (Figure 2B), 1.35 g·100 mL^−1^ for Co-3 (Figure 2C), and 4.69 g·100 mL^−1^ for Co-48 (Figure 2D), using culture medium 2 for Co-2 and Co-3 and culture medium 4 for Co-48. Among all co-culture conditions, Co-48 exhibited the highest biomass production. In this condition, the culture medium composition consisted of 10 g·L^−1^ glucose and 5 g·L^−1^ sucrose as carbon sources, 5 g·L^−1^ protein hydrolysate and 1 g·L^−1^ ammonium sulfate as nitrogen sources, and pH 5.5.

Conidial, microsclerotia, and chlamydospore counts were performed at the end of fermentation after 96 h, as shown in Table 1 and Table 2 for the co-cultures Co-2, Co-3, and Co-48. In the Co-2 dual culture between URM and IB, medium 1 showed the highest conidia·mL^−1^ at 25% URM and 75% IB. Medium 2 resulted in the highest microsclerotia·mL^−1^ at 75% URM and 25% IB, and medium 3 showed the highest chlamydospore·mL^−1^ at 50% URM and 50% IB. For the Co-2 dual culture between URM and MMBF, the highest conidia·mL^−1^ were observed at 50% URM and 50% MMBF in medium 2 and at 25% URM and 75% MMBF in medium 1. The highest microsclerotia·mL^−1^ were obtained in medium 1, and the highest chlamydospore·mL^−1^ in medium 2, both at 25% URM and 75% MMBF.

In the Co-3 co-culture, no microsclerotia were detected. Medium 4 showed the highest conidia·mL^−1^, while medium 3 resulted in the highest chlamydospores·mL^−1^, both at proportions of 16.7% MMBF, 66.7% IB, and 16.7% URM.

The triple co-culture with the addition of the third strain after 48 h (Co-48) showed no microsclerotia. The highest conidia·mL^−1^ were observed in medium 2 with the addition of MMBF at 48 h, while the highest chlamydospores·mL^−1^ were obtained in medium 3 with the addition of IB at 48 h. Comparing the three co-culture systems, culture medium 4 in Co-48 resulted in the highest biomass per 100 mL. The highest conidia and microsclerotia were observed in the Co-2 co-culture between URM and IB using media 1 and 2, respectively, while the highest chlamydospores were obtained in Co-3 using culture medium 3.

### 3.3. Quantitative Analysis of Growth-Promoting Activities

**Figure 3:**
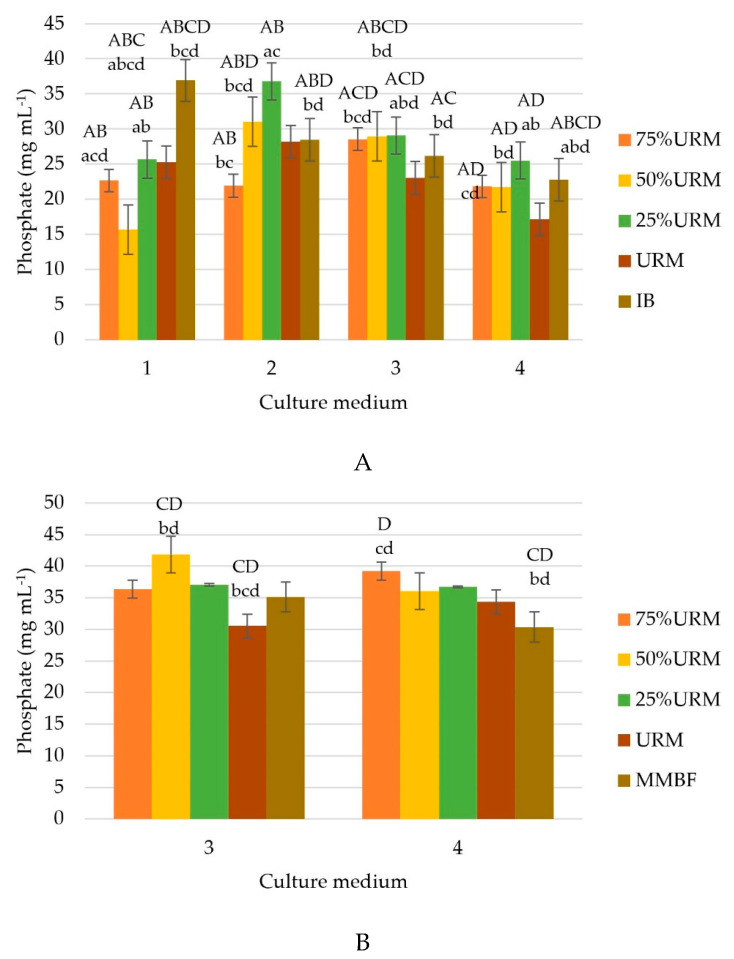
Phosphate solubilization in the dual co-culture of *Trichoderma* strains: (**A**) *T. harzianum* IB 19/17 and *T. asperellum* URM 6997/160821, and (**B**) *T. harzianum* MMBF 58/09 and *T. asperellum* URM 6997/160821. Different uppercase letters (A–D) indicate significant differences among culture media, whereas different lowercase letters (a–d) indicate differences among spore ratios (25%, 50%, 75% and unique culture, respectively), according to Tukey’s test (*p* < 0.05). Graphs without letter annotations showed no statistically significant differences according to Tukey’s test (*p* < 0.05).

Figure 3A,B show the quantification of phosphate solubilization for Co-2, since Co-3 and Co-48 did not exhibit phosphate solubilization. The highest quantified value for the Co-2 co-culture between URM and IB was obtained in fermentation using culture medium 2, reaching 25.7 mg·mL^−1^ at 25% URM and 75% IB. For the Co-2 co-culture between URM and MMBF, the highest value was observed in culture medium 3, with 41.9 mg·mL^−1^ at 50% URM and 50% MMBF. Comparing these two highlighted results, the co-culture between URM and MMBF showed the highest phosphate solubilization when both inoculants were used at equal proportions.

**Figure 4:**
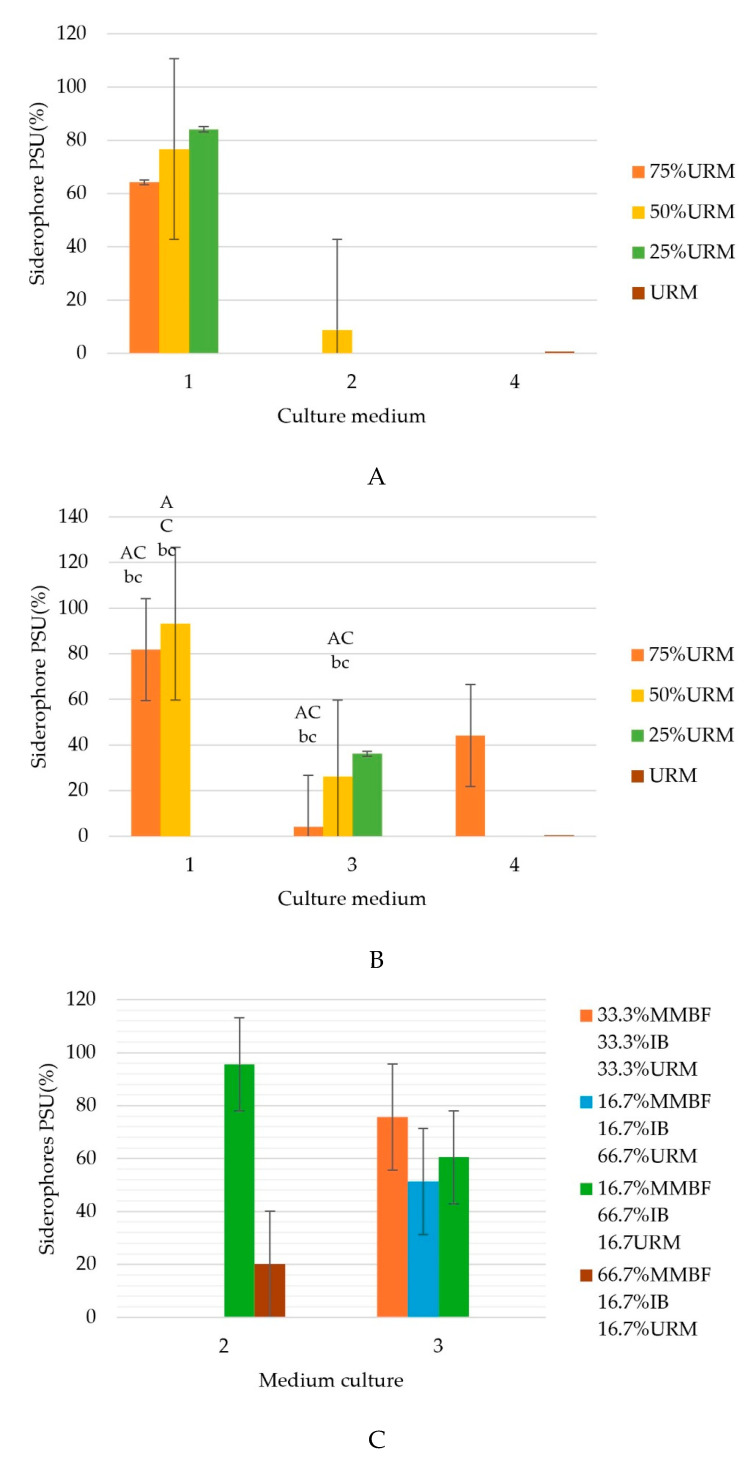

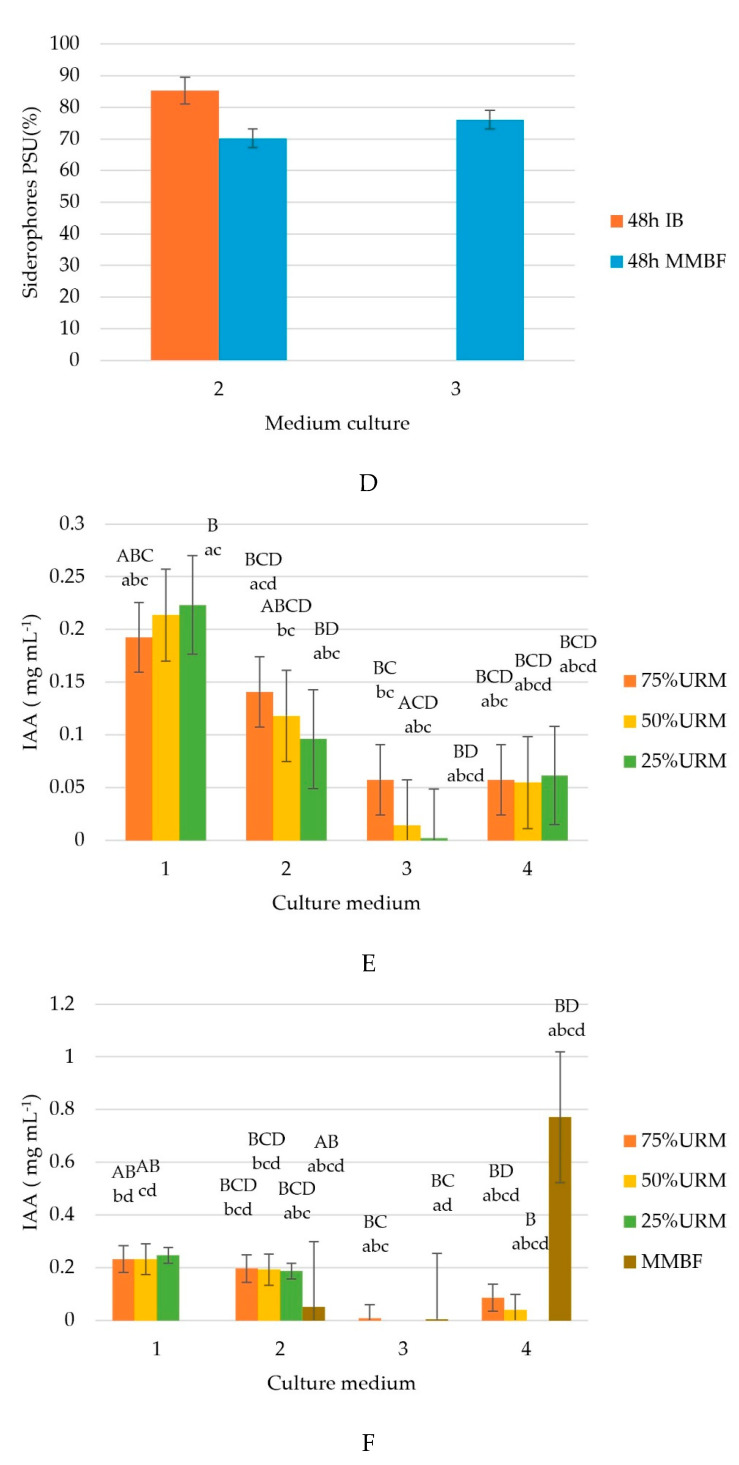

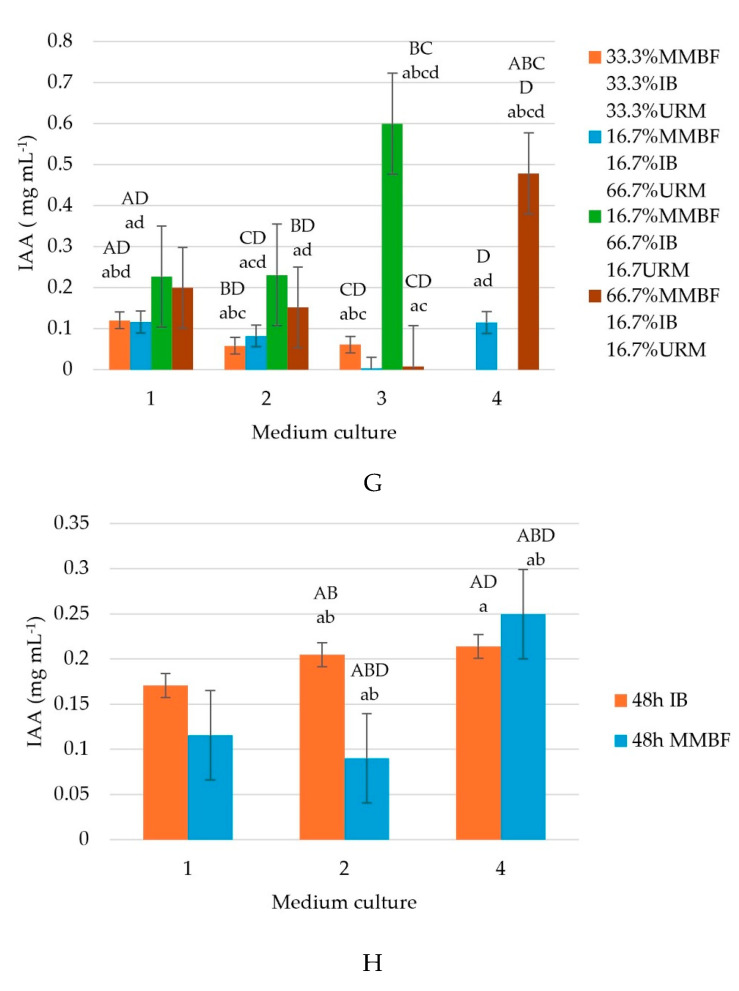
Siderophore (**A**–**D**) and IAA (**E**–**H**) production: (**A**,**E**) in Co-2 with *T. harzianum* IB 19/17 and *T. asperellum* URM 6997/160821; (**B**,**F**) in Co-2 with *T. harzianum* MMBF 58/09 and *T. asperellum* URM 6997/160821; (**C**,**G**) in Co-3; and (**D**,**H**) in Co-48. Different uppercase letters (A–D) indicate significant differences among culture media, whereas different lowercase letters (a–d) indicate differences among spore proportions, according to Tukey’s test (*p* < 0.05). Spore proportions were 25%, 50%, 75% and unique culture for Co-2, and 16.7%, 33.3%, and 66.7% for Co-3 and Co-48. In the Co-48 treatment, the third strain was added 48 h after the beginning of fermentation. Graphs without letter annotations showed no statistically significant differences according to Tukey’s test (*p* < 0.05).

All three co-culture systems showed siderophore production. In the Co-2 co-culture between URM and IB (Figure 4A), the highest production was observed in culture medium 1, with 25% URM and 75% IB, reaching 84%. Similarly, in the Co-2 co-culture between URM and MMBF (Figure 4B), the highest siderophore production occurred in culture medium 1 at 50% URM and 50% MMBF, reaching 93%. These values were lower than those observed in the monocultures of both URM and IB, in which no siderophore production was detected.

The Co-3 co-culture (Figure 4C) showed the highest siderophore production in culture medium 2, with 16.7% MMBF, 16.7% URM, and 66.7% IB, reaching 95%. When comparing the co-culture systems, Co-3 exhibited the highest siderophore production overall. For Co-48 (Figure 4D), the highest production was observed in culture medium 2, with the addition of the IB strain at 48 h, reaching 85%.

Culture medium 1 yielded the best results for Co-2, whereas culture medium 2 was more favorable for Co-3 and Co-48. The main differences between these media are related to the nitrogen source and pH: medium 1 contains 2 g·L^−1^ protein hydrolysate and 3 g·L^−1^ ammonium sulfate at pH 4.5, while medium 2 contains 8 g·L^−1^ protein hydrolysate at pH 6.5.

In the analysis of co-cultures for IAA production, all three fermentation systems showed IAA production, with 0.22 mg·mL^−1^ for the Co-2 co-culture between URM and IB (Figure 4E) and 0.25 mg·mL^−1^ for the Co-2 co-culture between URM and MMBF (Figure 4F), both obtained using culture medium 1. The Co-3 co-culture (Figure 4G) showed the highest IAA production, reaching 0.60 mg·mL^−1^ with 16.7% MMBF, 16.7% URM, and 66.7% IB in culture medium 3. For Co-48 (Figure 4H), IAA production reached 0.25 mg·mL^−1^ in culture medium 4. When comparing the co-culture systems, similarly to siderophore production, the Co-3 co-culture showed the highest IAA production. In this condition, the culture medium contained low concentrations of carbon (5 g·L^−1^ glucose) and nitrogen (2 g·L^−1^ protein hydrolysate and 3 g·L^−1^ ammonium sulfate) at pH 6.5.

### 3.4. Enzymatic Assays

**Figure 5:**
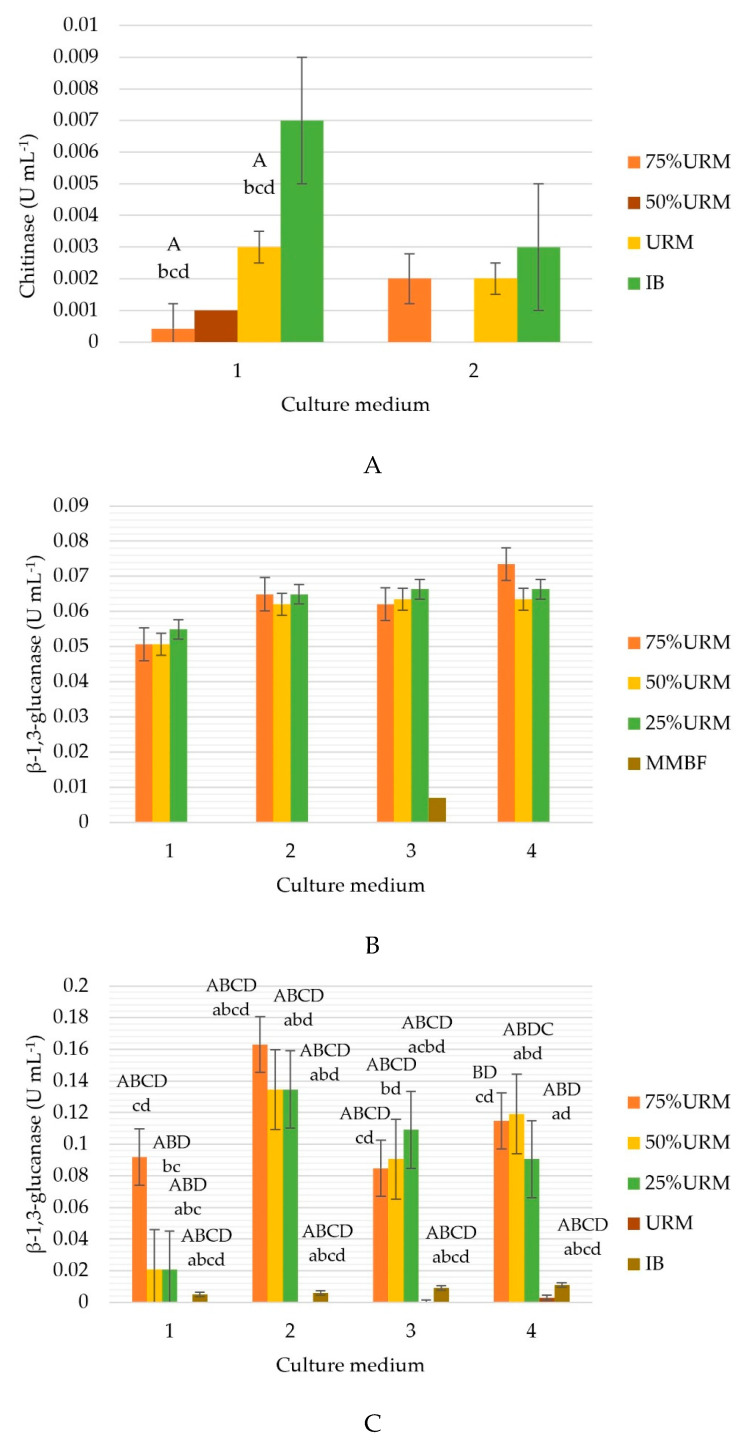

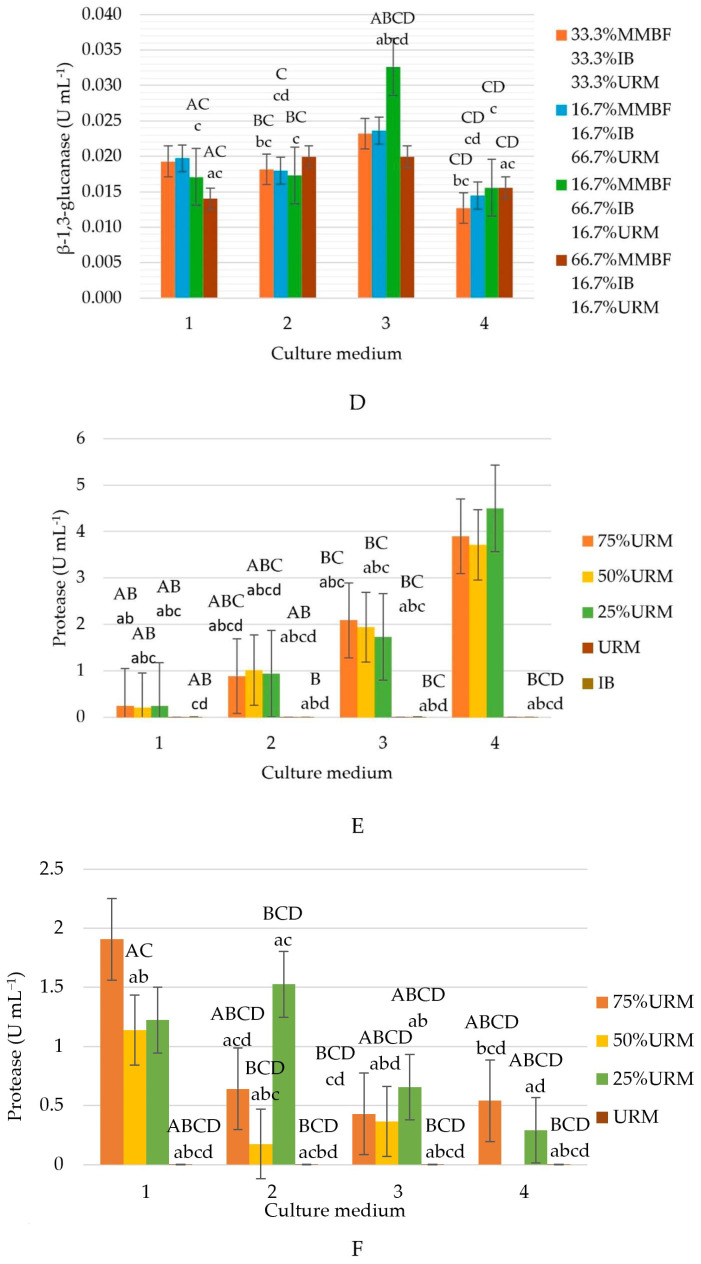

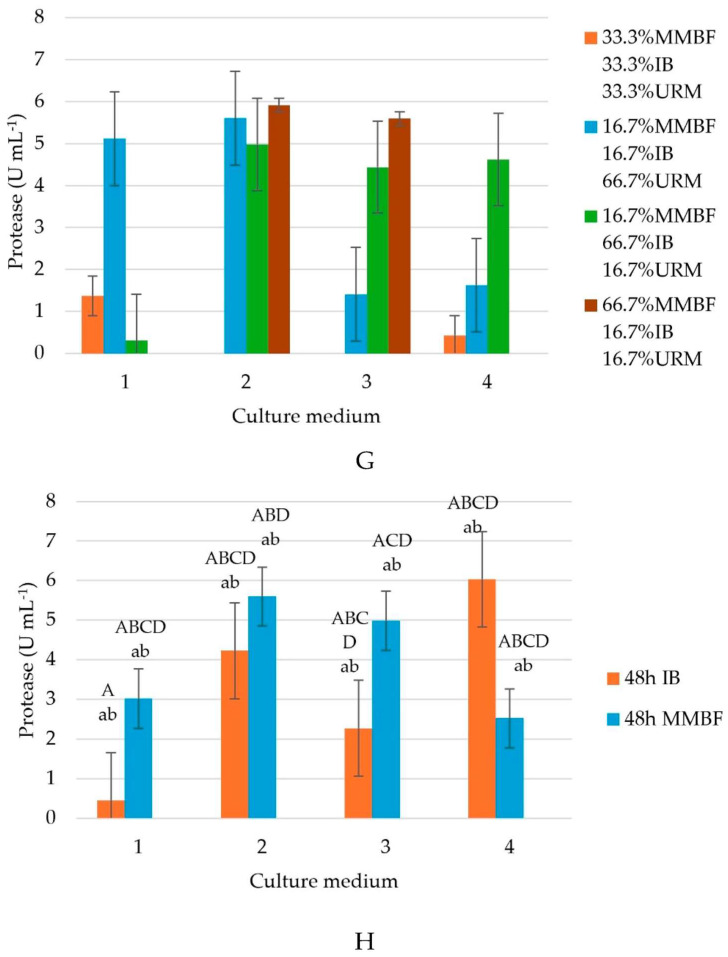
Chitinase (**A**), β-1,3-glucanase (**B**–**D**) and protease (**E**–**H**) activities: (**A**,**B**,**E**) Co-2 with *T. harzianum* IB 19/17 and *T. asperellum* URM 6997/160821; (**C**,**F**) Co-2 with *T. harzianum* MMBF 58/09 and *T. asperellum* URM 6997/160821; (**D**,**G**) in Co-3; and (**H**) in Co-48. Different uppercase letters (A–D) indicate significant differences among culture media, whereas different lowercase letters (a–d) indicate differences among spore proportions, according to Tukey’s test (*p* < 0.05). Spore proportions were 25%, 50%, 75% and unique culture for Co-2, and 16.7%, 33.3%, and 66.7% for Co-3 and Co-48. In the Co-48 treatment, the third strain was added 48 h after the beginning of fermentation. Graphs without letter annotations showed no statistically significant differences according to Tukey’s test (*p* < 0.05).

Only Co-2 co-culture showed quantifiable chitinase activity, with 75% URM and 25% IB (Figure 5A), reaching 0.002 U·mL^−1^ in culture medium 2. This value was lower than that observed in the IB monoculture (0.007 U·mL^−1^). The other co-culture conditions, Co-2 between URM and MMBF, Co-3, and Co-48, did not show detectable chitinase activity.

β-1,3-glucanase enzymatic activity was quantified in the Co-2 co-cultures (Figure 5B,C) and in Co-3 (Figure 5D), with no detectable activity in Co-48. The Co-2 co-culture between URM and IB showed an activity of 0.16 U·mL^−1^ in culture medium 2. The Co-2 co-culture between URM and MMBF presented 0.073 U·mL^−1^ in culture medium 4, while Co-3, at 16.7% MMBF, 16.7% URM, and 66.7% IB, showed 0.032 U·mL^−1^ of enzymatic activity in culture medium 3. When comparing the co-culture systems, the highest β-1,3-glucanase activity was observed in the Co-2 co-culture between URM and IB. In this condition, the culture medium contained 10 g·L^−1^ glucose and 5 g·L^−1^ sucrose as carbon sources, 5 g·L^−1^ protein hydrolysate and 1 g·L^−1^ ammonium sulfate as nitrogen sources, and pH 5.5.

Protease activity was detected in all three co-culture systems. In the Co-2 co-culture between URM and IB (Figure 5E), the highest activity was observed in culture medium 4 at 25% URM and 75% IB, reaching 4.50 U·mL^−1^. For the Co-2 co-culture between URM and MMBF (Figure 5F), protease activity reached 1.91 U·mL^−1^ in culture medium 1 at 75% URM and 25% MMBF. The Co-3 co-culture (Figure 5G) showed protease activity of 5.92 U·mL^−1^ in culture medium 1 with 66.7% MMBF and 16.7% URM and IB. In Co-48 (Figure 5H), protease activity reached 6.0 U·mL^−1^, representing the highest protease activity among the co-culture systems.

### 3.5. Gas Chromatography–Mass Spectrometry

Table 3 presents the comparative GC-MS identification of compounds, highlighting the metabolic plasticity of the endophytic Trichoderma strains under different fermentation conditions. Across the seven fermentations, the number of identifiable compounds varied notably, ranging from a minimum of 5 to a maximum of 20 depending on the growth conditions. In general, the co-cultures exhibited a higher number of metabolites across the different fermentation media compared to the individual isolates. The chemical output was consistently dominated by fatty acid derivatives—specifically 13-Docosenamide, 9-Octadecenamide, and Octadecanoic acid—alongside various long-chain alcohols and alkanes. While these core metabolites were prevalent, specific strains exhibited distinct production signatures, such as the unique expression of 6-Amyl alpha pyrone by strain Co-3 in Medium 4. Overall, these results indicate that fermentation medium composition is a primary driver of metabolite diversity in Trichoderma endophytes.

## 4. Discussion

Microbial co-culture systems can be viewed as experimental analogues of naturally occurring multispecies interactions in soil and rhizosphere environments, where cooperation and competition regulate microbial functioning. Co-cultivation has been reported to promote plant growth and development by enhancing microbial interactions and metabolic complementarity [11]. Interactions between mycorrhizal fungi and filamentous endophytic fungi, such as *G. margarita* and *T. harzianum*, have been shown to stimulate plant growth and increase the availability and solubilization of nutrients, including P and Zn, in bean crops [23].

Microbial compatibility is a prerequisite for the successful establishment of multi-strain inoculants in soil environments, where spatial overlap and resource competition are inevitable. The absence of antagonistic interactions among the tested *Trichoderma* strains suggests their potential to coexist within soil niches, supporting functional redundancy and resilience in microbial communities [24]. In compatibility assays between *Pseudomonas protegens* and *Trichoderma* spp., no antagonistic activity was observed between the bacterium and the fungus, indicating the viability of these strains for application in plant growth promotion [10]. In the present study, only the mixture of spores from URM and MMBF inoculated at the center of the plate with sucrose as the carbon source failed to sporulate. All other combinations showed visual compatibility.

In the evaluation of compatibility between *Trichoderma reesei* and *Aspergillus* species (*Aspergillus saccharolyticus*, *A. carbonarius*, and *A. niger*) on plates containing different carbon sources, such as glucose, carboxymethylcellulose (CMC), and Avicel, partial compatibility was observed. The microorganisms were able to grow around each other on glucose-containing plates, whereas only *T. reesei* and *A. niger* exhibited compatibility on Avicel, representing the microbial combination with the highest number of interactions. When mixed spores were spread on Avicel, *T. reesei* was inhibited by *A. saccharolyticus* and *A. carbonarius*, while CMC showed the lowest level of compatibility among the tested substrates [10].

The observed differences in biomass production among co-culture systems reflect the sensitivity of *Trichoderma* species to nutrient availability, a characteristic that mirrors the spatial and temporal heterogeneity of soil environments. Balanced carbon and nitrogen inputs favored microbial growth, suggesting that similar nutrient conditions in soil may promote the establishment and persistence of these fungi. As reported by Taskin et al. (2012) [25], an adequate carbon-to-nitrogen ratio is essential, as it directly influences cellular growth and metabolic activity, such as lactic acid synthesis in *Rhizopus* strains.

Both growth and metabolic performance of *Trichoderma* are strongly dependent on nutritional conditions, particularly carbon and nitrogen sources, which directly affect sporulation, in addition to environmental factors such as pH, temperature, and light incidence. For example, studies with *T. afroharzianum* and *T. asperelloides* demonstrated higher spore production when soluble starch and maltose were used as carbon sources, respectively, and peptone as the nitrogen source, whereas growth was impaired when urea was used as the nitrogen source [26].

Different *Trichoderma* species exhibit rapid growth, extensive substrate assimilation, and the potential to produce diverse microbial agents. They synthesize secondary metabolites, such as siderophores, which possess antimicrobial properties, including antibacterial activity, contributing to the suppression of soil-borne phytopathogens through iron sequestration and inactivation of iron-dependent enzymes. Another key function of *Trichoderma* is the production of IAA and the enhancement of phosphorus bioavailability, as phytase enzymes catalyze the conversion of insoluble soil phosphate, thereby improving nutrient uptake by plants [27].

High siderophore production suggests a strong capacity for iron mobilization in soil environments, contributing to competitive regulation of microbial competition and improved micronutrient acquisition by plants [28]. The high siderophore levels observed in specific co-culture conditions indicate an enhanced capacity for iron mobilization, which may contribute to the suppression of soil-borne pathogens and influence rhizosphere microbial dynamics.

Fungi predominantly synthesize hydroxamate-type siderophores derived from the non-proteinogenic amino acid ornithine. Species of *Trichoderma* have been reported as prolific siderophore producers [29]. Both *T. harzianum* and *T. viride* have been shown to produce IAA, siderophores, and solubilize phosphate [30]. Furthermore, in melon, pepper, and tomato crops, *T. viride* produces hydroxamate- and catechol-type siderophores [31].

Phosphorus (P) is a key macronutrient for plant development and growth. Plants absorb P primarily as soil orthophosphate (H_2_PO_4_^−^ or HPO_4_^2−^) at micromolar concentrations, and it is a component of numerous biomolecules, including nucleic acids, enzymes, coenzymes, phosphoproteins, and phospholipids. Synthetic fertilizers are typically produced by acidifying phosphate rock, a slow, resource-intensive process that generates environmental pollution and can lead to nutrient losses [27,32].

Microorganisms capable of phosphate solubilization, such as fungi, facilitate the release of P through the production of organic acids, including citric, gluconic, oxalic, propionic, formic, succinic, acetic, and lactic acids, as well as siderophores and exopolysaccharides. When applied to soil, these fungi increase the availability of P by mobilizing phosphate bound to soil particles through interactions with Ca^2+^, Fe^3+^, and Al^3+^ ions^.^ [32].

Fungi generally exhibit higher inorganic phosphate solubilization than bacteria, due to their deeper soil penetration and higher production of organic acids. Several fungal genera, including *Aspergillus*, *Fusarium*, *Penicillium*, and *Trichoderma*, can mobilize phosphate for crops such as cabbage, fava bean, sugarcane, and tomato. Many species, including *A. niger*, *T. harzianum*, and *T. viride*, produce phytase, enabling the release of phosphate from phytic acid [33].

Phosphate solubilization by *Trichoderma* co-cultures highlights their functional relevance in soil nutrient cycling. By mobilizing inorganic phosphate through organic acid production and associated mechanisms, these fungi can increase phosphorus availability in soils with low P bioaccessibility, reducing dependence on synthetic fertilizers [34].

Different *Trichoderma* isolates have been reported to solubilize phosphate and produce IAA while controlling *Fusarium* wilt in banana plants [35]. In the present study, cocultures of *Trichoderma* strains demonstrated efficient phosphate solubilization, with Co-2 (URM/MMBF, 50:50) achieving 419 mg·mL^−1^, underscoring their potential for enhancing nutrient availability, plant growth, and biocontrol.

IAA, a phytohormone of the auxin family, regulates plant growth by promoting root elongation, cell division, and expansion [36]. Microbial production of IAA contributes to plant-microbe interactions and can modulate the surrounding microbial community, enhancing plant immunity. While moderate IAA levels favor plant development, elevated concentrations may indicate the presence of fungal or bacterial phytopathogens [37]. *Trichoderma* species, including *T. virens*, *T. koningii*, and *T. harzianum*, promote biocontrol of *Sclerotinia sclerotiorum* and *Rhizoctonia solani* in common bean (*Phaseolus vulgaris*) while stimulating the production of hydrolases, IAA, cytokinins, gibberellins, and siderophores [30].

*Trichoderma* spp. is well-known for producing antimicrobial compounds and are widely used as biocontrol agents. They can synthesize resistance-related enzymes such as chitinase, β-1,3-glucanase, and protease, as well as phenolic compounds, enhancing systemic resistance in plants and improving soil enzymatic activity. These enzymes are central to organic matter decomposition and pathogen suppression in soils [38]. The enzymatic profiles observed in the present study suggest that co-cultured *Trichoderma* strains may contribute to soil biochemical activity by accelerating residue turnover and limiting the proliferation of phytopathogenic fungi.

Co-cultivation with other microorganisms, such as *T. harzianum* with the cyanobacterium *Spirulina maxima*, which fixes atmospheric nitrogen, solubilizes phosphate, and produces amino acids, polypeptides, siderophores, and bioactive compounds, has been shown to positively regulate defense-related genes, including JERF3 (a transcription factor modulating multiple stress-responsive genes), peroxidase, and chitinase, enhancing plant resistance [29].

In co-cultivation of *T. harzianum* with *F. oxysporum*, fungal proteases have several agricultural applications, contributing to seed treatment, enhanced nutrient bioavailability, reduced soil toxicity, and overall soil sustainability. Some hydrolases are involved in phosphate and potassium solubilization and in the production of plant growth-promoting hormones [39].

Secondary metabolites produced by *Trichoderma* species, including diverse terpenoids, are ecologically relevant in soil and rhizosphere environments, where they modulate microbial interactions, contribute to pathogen suppression, and influence plant–soil feedbacks, highlighting their functional role in soil ecosystem processes [40]. Beyond their antimicrobial properties, secondary metabolites produced by *Trichoderma* may also function as signaling molecules that affect plant–microbe communication in the rhizosphere. By modulating root-associated microbial assemblages and interacting with plant signaling pathways, these compounds can indirectly influence root development, nutrient uptake efficiency, and rhizosphere biochemical activity. Consequently, metabolite diversity contributes to feedback mechanisms that link microbial metabolism with plant performance and soil nutrient dynamics [41].

Overall, the production of secondary metabolites by *Trichoderma* species reinforces their role as functional components. These compounds contribute to key services, including biological control of soil-borne pathogens, enhancement of rhizosphere functioning, and support of sustainable nutrient cycling. From a technological standpoint, the enhanced enzymatic and metabolite production observed under co-fermentation conditions indicates a strong potential for process intensification and scale-up in submerged fermentation systems. The robustness of the co-culture strategy across different media compositions suggests its applicability in industrial bioprocesses aimed at producing multifunctional bioinputs for sustainable agriculture.

## 5. Conclusions

This study demonstrates that co-fermentation of *Trichoderma* strains constitutes an effective biotechnological strategy to intensify the production of enzymes and bioactive metabolites relevant for plant assays. The modulation of culture media composition and inoculation strategies significantly enhanced phosphate solubilization, siderophore and phytohormone synthesis, hydrolytic enzyme activities, and secondary metabolite diversity. These results highlight the potential of microbial co-culture systems to promote metabolic complementarity and improve biocatalytic efficiency under controlled fermentation conditions. From a technological perspective, *Trichoderma* co-fermentation offers a promising platform for the development of multifunctional bio-based inputs, combining nutrient mobilization, biocontrol potential, and plant growth promotion, thereby contributing to more sustainable and efficient agricultural bioprocesses.

## Figures and Tables

**Table 1:** Propagules yields for the dual co-culture (Co-2) at proportions of 25%, 50%, and 75% among the three strains *T. harzianum* IB 19/17, *T. asperellum* URM 6997/160821, and *T. harzianum* MMBF 58/09.

Culture Medium	Sample	Conidia	Microsclerotia	Chlamydospores
1	75% URM 25% IB	335 × 10^4^	10 × 10^4^	525 × 10^4^
	50% URM/IB	510 × 10^4^	20 × 10^4^	113 × 10^4^
	25% URM 75% IB	1835 × 10^4^	15 × 10^4^	393 × 10^4^
	75% URM 25% MMBF	110 × 10^4^	-	90 × 10^4^
	50% URM/MMBF	80 × 10^4^	2.5 × 10^4^	115 × 10^4^
	25% URM 75% MMBF	210 × 10^4^	12.5 × 10^4^	163 × 10^4^
	100% URM	425 × 10^4^	-	40 × 10^4^
	100% IB	190 × 10^4^	-	-
	100% MMBF	25 × 10^4^	-	17.5 × 10^4^
2	75% URM 25% IB	310 × 10^4^	32.5 × 10^4^	50 × 10^4^
	50% URM/IB	185 × 10^4^	15 × 10^4^	32.5 × 10^4^
	25% URM 75% IB	205 × 10^4^	12.5 × 10^4^	128 × 10^4^
	75% URM 25% MMBF	145 × 10^4^	-	57.5 × 10^4^
	50% URM/MMBF	210 × 10^4^	-	180 × 10^4^
	25% URM 75% MMBF	70 × 10^4^	-	203 × 10^4^
	100% URM	135 × 10^4^	5 × 10^4^	135 × 10^4^
	100% IB	1530 × 10^4^	10 × 10^4^	143 × 10^4^
	100% MMBF	765 × 10^4^	-	40 × 10^4^
3	75% URM 25% IB	190 × 10^4^	10 × 10^4^	753 × 10^4^
	50% URM/IB	15 × 10^4^	5 × 10^4^	1150 × 10^4^
	25% URM 75% IB	5 × 10^4^	2.5 × 10^4^	500 × 10^4^
	75% URM 25% MMBF	160 × 10^4^	-	57.5 × 10^4^
	50% URM/MMBF	40 × 10^4^	-	30 × 10^4^
	25% URM 75% MMBF	50 × 10^4^	-	50 × 10^4^
	100% URM	110 × 10^4^	2.5 × 10^4^	908 × 10^4^
	100% IB	25 × 10^4^	7.5 × 10^4^	1005 × 10^4^
	100% MMBF	3750 × 10^4^	-	-
4	75% URM 25% IB	65 × 10^4^	2.5 × 10^4^	230 × 10^4^
	50% URM/IB	20 × 10^4^	7.5 × 10^4^	265 × 10^4^
	25% URM 75% IB	35 × 10^4^	32.5 × 10^4^	180 × 10^4^
	75% URM 25% MMBF	-	-	17.5 × 10^4^
	50% URM/MMBF	35 × 10^4^	-	125 × 10^4^
	25% URM 75% MMBF	10 × 10^4^	5 × 10^4^	27.5 × 10^4^
	100% URM	15 × 10^4^	-	655 × 10^4^
	100% IB	10 × 10^4^	5 × 10^4^	863 × 10^4^
	100% MMBF	40 × 10^4^	-	30 × 10^4^

**Table 2:** Conidia and chlamydospores production for the triple co-culture at proportions of 16.7%, 33.3%, and 66.7% (Co-3), and with the addition of the third strain after 48 h at a proportion of 33.3% (Co-48), for each of the three strains *T. harzianum* IB 19/17, *T. asperellum* URM 6997/160821, and *T. harzianum* MMBF 58/09.

Culture Medium	Sample	Conidia	Chlamydospores
Co-3			
1	33.3% MMBF/IB/URM	75 × 10^4^	-
	16.7% MMBF/IB 66.7% URM	250 × 10^4^	-
	16.7% MMBF/URM 66.7% IB	75 × 10^4^	-
	16.7% IB/URM 66.7% MMBF	425 × 10^4^	40 × 10^4^
2	33.3% MMBF/IB/URM	25 × 10^4^	-
	16.7% MMBF/IB 66.7% URM	550 × 10^4^	-
	16.7% MMBF/URM 66.7% IB	250 × 10^4^	-
	16.7% IB/URM 66.7% MMBF	475 × 10^4^	-
3	33.3% MMBF/IB/URM	75 × 10^4^	470 × 10^4^
	16.7% MMBF/IB 66.7% URM	325 × 10^4^	50 × 10^4^
	16.7% MMBF/URM 66.7% IB	50 × 10^4^	1570 × 10^4^
	16.7% IB/URM 66.7% MMBF	125 × 10^4^	470 × 10^4^
4	33.3% MMBF/IB/URM	75 × 10^4^	50 × 10^4^
	16.7% MMBF/IB 66.7% URM	375 × 10^4^	-
	16.7% MMBF/URM 66.7% IB	575 × 10^4^	10 × 10^4^
	16.7% IB/URM 66.7% MMBF	450 × 10^4^	-
Co-48			
1	48 h IB	125 × 10^4^	-
	48 h MMBF	125 × 10^4^	-
2	48 h IB	375 × 10^4^	-
	48 h MMBF	475 × 10^4^	-
3	48 h IB	100 × 10^4^	850 × 10^4^
	48 h MMBF	225 × 10^4^	680 × 10^4^
4	48 h IB	225 × 10^4^	-
	48 h MMBF	175 × 10^4^	-

**Table 3:** Comparative GC-MS Profile of Secondary Metabolites Produced by Endophytic Trichoderma Strains.

	Medium 1		Medium 2		Medium 3		Medium 4	
Fermentation	Number of Compounds	* Main Compounds	Number of Compounds	Main Compounds	Number of Compounds	Main Compounds	Number of Compounds	Main Compounds
Co-2 IB	18	1-Hexanol, 2-ethyl-	14	1-Hexanol, 2-ethyl-	12	9-Octadecenamide	15	9-Octadecenamide
		9-Octadecenamide		9-Octadecenamide		Undecane		o-Cymene
		Undecane		Tetradecane		Tetradecane		Guaiol
Co-2 MMBF	7	13-decosenamide, (Z)	9	13-decosenamide, (Z)	8	13-decosenamide, (Z)	9	13-Docosenamide, (Z)
		Tetradecane		1-Hexanol, 2-ethyl-		1-Hexanol, 2-ethyl-		Tetracontane
		Dodecane		Dodecane		Tridecane		1-Hexanol, 2-ethyl-
Co-3	16	n-Hexadecanoic acid	12	1-Hexanol, 2-ethyl-	12	13-decosenamide, (Z)	20	6 Amyl alpha pyrone
		Oleic Acid		Undecane		Undecane		13-decosenamide, (Z)
		Octadecanoic acid		n-Hexadecanoic acid		1-Hexanol, 2-ethyl-		Docosane
Co-48	16	1-Hexanol, 2-ethyl	19	Phenylethyl Alcohol	14	13-Docosenamide, (Z)	15	1-Hexanol, 2-ethyl-
		Octadecanoic acid		1-Hexanol, 2-ethyl-		Dodecane		9-Octadecenamide, (Z)
		13-Docosenamide, (Z)		Undecane		Undecane		Tridecane
IB	12	13-Docosenamide, (Z)-	11	Hexadecane	7	Hexadecane	8	9-Octadecenamide
		Octadecanoic acid		9-Octadecenamide		Tetradecane		Tetradecane
		Dodecane		Tetradecane		9-Octadecenamide		Octadecanoic acid
URM	16	13-Docosenamide, (Z)-	5	1-Hexanol, 2-ethyl-	9	Docosane	7	Docosane
		1-Hexanol, 2-ethyl-		Docosane		9-Octadecenamide, (Z)-		1-Hexanol, 2-ethyl-
		Octadecanoic acid		Octadecanoic acid		n-Hexadecanoic acid		n-Hexadecanoic acid
MMBF	10	9-Octadecenoic acid (Z)-, methyl ester	8	Tridecane	11	Docosane	6	9-Octadecenoic acid (Z)-, methyl ester
		Docosane		Docosane		Dotriacontane		Dotriacontane
		Tridecane		9-Octadecenoic acid (Z)-, methyl ester		Tetradecane		Docosane

* Only the three main compounds (based on relative are of picks) were presented.

## Data Availability

The original contributions presented in this study are included in the article. Further inquiries can be directed to the corresponding author.
